# Supplementary figures and images for: Genetic modulation of mitochondrial NAD+ regeneration does not prevent dopaminergic neuron dysfunction caused by mitochondrial complex I impairment
Source: Front Cell Dev Biol. 2025 Sep 25;13:1650462. doi: 10.3389/fcell.2025.1650462 (PMC12507863; doi:10.3389/fcell.2025.1650462)

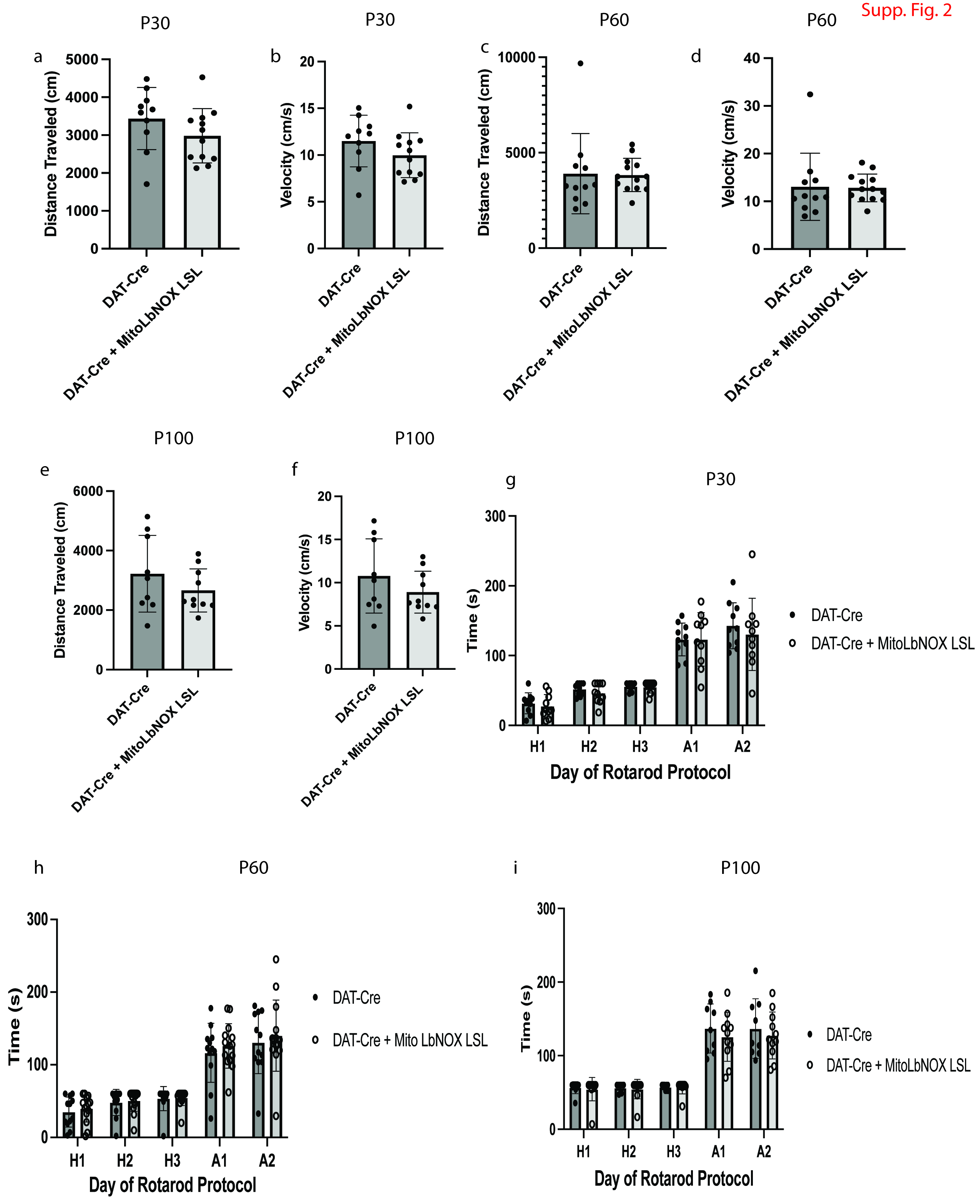

Supplement: Supplementary file 1 [file Image2.tif]

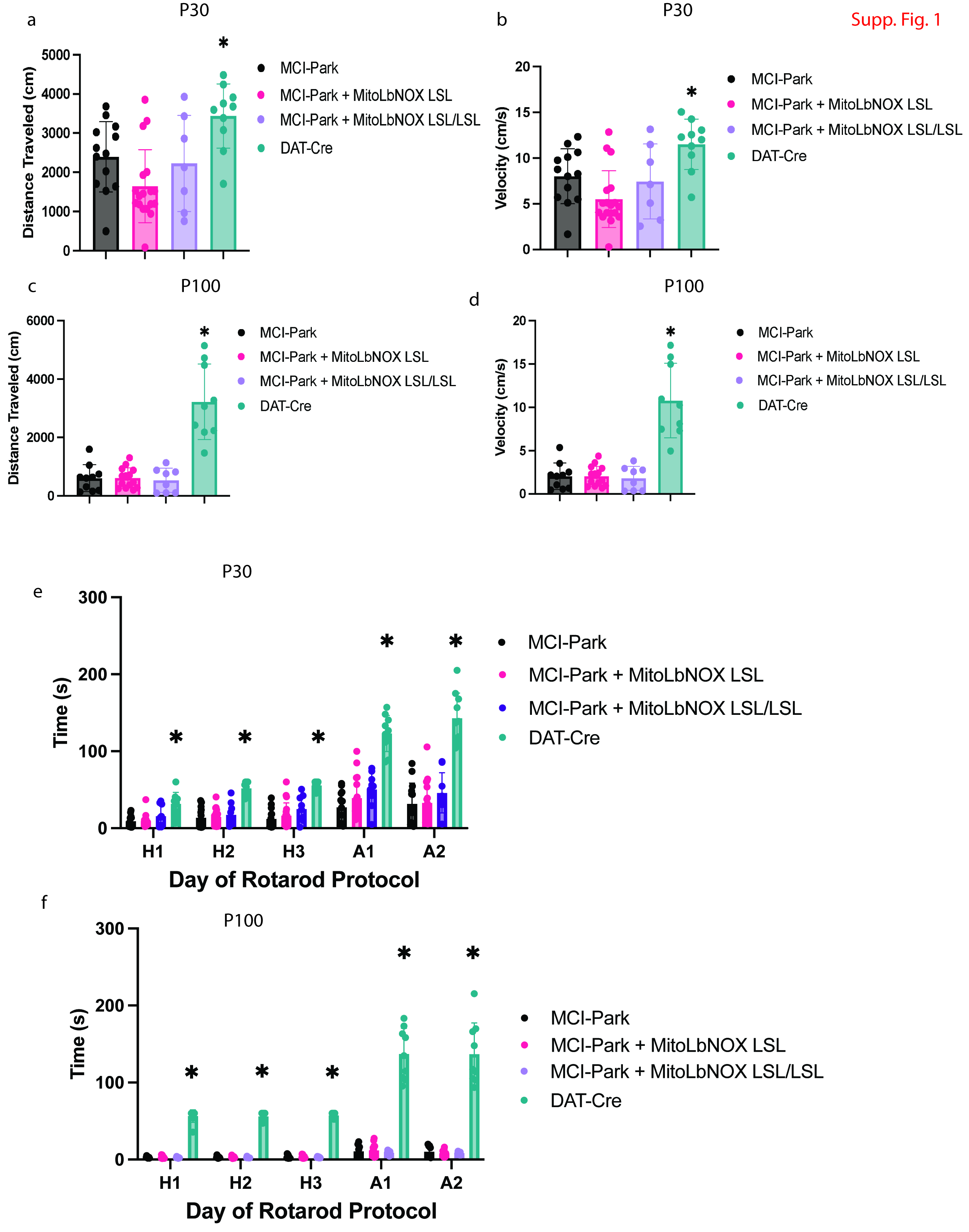

Supplement: Supplementary file 2 [file Image1.tif]
